# *Coriobacteriia* may affect the occurrence of hepatocellular carcinoma through the pyrimidine, caffeine, urea cycle and glutamate metabolic pathways

**DOI:** 10.1097/MD.0000000000045663

**Published:** 2025-11-21

**Authors:** Jingyi Dai, Qiujing Li, Jie Chen, Guiming Liu, Zhijian Dong, Zhongxu Ma, Yu Luo

**Affiliations:** a Department of Liver Diseases, The Third People’s Hospital of Kunming City/Infectious Disease Clinical Medical Center of Yunnan Province, Kunming, China; b Department of Public Laboratory, The Third People’s Hospital of Kunming City/ Infectious Disease Clinical Medical Center of Yunnan Province, Kunming, China; c Department of Pharmacy, The Third People’s Hospital of Kunming City/Infectious Disease Clinical Medical Center of Yunnan Province, Kunming, China; d Department of Public Laboratory, The Third People’s Hospital of Kunming City/Infectious Disease Clinical Medical Center of Yunnan Province, Kunming, China; e Department of Ultrasonics, The Third People’s Hospital of Kunming City/Infectious Disease Clinical Medical Center of Yunnan Province, Kunming, China; f Department of Internal Medicine, The Third People’s Hospital of Kunming City/Infectious Disease Clinical Medical Center of Yunnan Province, Kunming, China.

**Keywords:** *Coriobacteriia*, correlations, gut microbiota, hepatocellular carcinoma, intermediary factor, Mendelian randomization, metabolite

## Abstract

Most patients with hepatocellular carcinoma (HCC) lose the chance of survival due to lack of timely diagnosis and effective treatment. The specific gut microbiota (GM) spectrum may become the target of early diagnosis and treatment of HCC. However, the metabolic mechanisms that affect the occurrence of liver cancer are still unclear. In this study, we called the dataset of HCC, and 1091 serum metabolites 309 metabolite ratios as well as 211 GM taxa through genome-wide association studies instrumental variables for Mendelian randomization causal association analysis and metabolome intermediary effects exploration. Through functional enrichment of intermediate metabolites, the effects of metabolic pathways involved in GM on HCC were analyzed. Inverse variance weighting was the main model for establishing causal associations. Additionally horizontal pleiotropy test, linkage-disequilibrium test and the sensitivity analysis were employed to test the explanatory power of instrumental variables (single nucleotide polymorphisms). Our study found Coriobacteriia class, Coriobacteriales order, Coriobacteriaceae family, and 4 specific genera were strongly related to HCC (*P* <.05). Meanwhile, through 2 samples-MR Analysis, 49 metabolites levels/ratios were shown to be closely related to the development of HCC. A total of 10 related metabolic intermediary factors have been selected, and 4 metabolic pathways of pyrimidine (*P* = .0031), caffeine (*P* = .0072), urea cycle (*P* = .0105) and glutamate (*P* = .0298) were significantly enriched in this GM related HCC process. *Coriobacteriia* class and its lower taxa were associated with the risk factors of developing HCC through the regulation of pyrimidine, caffeine, urea cycle and glutamate metabolic pathways. These GM taxa and metabolitesmay serve as potential targets for the diagnosis of HCC.

## 1. Introduction

Hepatocellular carcinoma (HCC) is a malignant tumor that originates in liver cells. The HCC accounts for 80% of primary liver cancer and is the third leading cause of cancer-related death.^[1,2]^ According to epidemiological surveys, 84,000 new cases of liver cancer were diagnosed in 2018, while the number of deaths has reached 78,000 cases, becoming one of the diseases with high clinical mortality.^[3]^ The primary risk factor for HCC is underlying cirrhosis, with approximately 90% of cases being attributed to hepatitis C virus (HCV), hepatitis B virus (HBV), alcohol-related liver disease steatosis (ALD), and metabolic dysfunction associated with liver disease (MASLD).^[4]^ The progression from viral hepatitis to cirrhosis to HCC is extremely complex and varies greatly from individual to individual. The pathogenesis involves a variety of internal and external factors. The early clinical manifestations of liver cancer are often inconspicuous, resulting in delayed diagnosis and ineffective treatment for over 2-thirds of patients, leading to a low survival rate. The currently available diagnostic markers are insufficient to meet the practical demands of clinical practice, thereby highlighting the urgent need for the discovery of novel, highly sensitive, and minimally invasive diagnostic markers.^[5]^

The influence of the gut environment on the liver is particularly important because of its close anatomical relationship with the liver. The gut and liver are intricately interconnected and communicate via the portal vein and biliary system, thereby exposing the liver to bacterial products and metabolites originating from the gut. The integrity of the intestinal barrier is crucial for maintaining a physical and functional separation between the gut and host-associated microbes. Disruption of barrier function can result in heightened bacterial translocation and leakage of bacterial metabolites.^[6]^ Recently, it has been observed that patients with cirrhosis and HCC exhibit a decreased diversity of gut microbiota (GM) compared to healthy controls.^[7]^ The specific composition of GM in HCC holds significant clinical diagnostic value. Furthermore, researchers have discovered that toll-like receptors mediate the impact of GM on liver fibrosis progression.^[8]^ These findings suggest the potential for modulating GM as a therapeutic approach for managing HCC.

The comprehension of the biological and chemical mechanisms that influence the progression of HCC is a crucial step in developing targeted prediction and treatment strategies. The alteration in serum metabolism serves as the primary mechanism through which GM interacts with the liver. Short-chain fatty acids (SCFA) exhibit potent anticancer properties, yet their synthesis tends to decrease in liver cancer patients due to microbial imbalance.^[9]^ Although the traditional perspective holds that SCFA are generally beneficial to health, recent studies have suggested that a diet high in inulin may induce cholestasis and contribute to the initiation and progression of HCC, potentially through the GM-mediated metabolism of SCFA derived from cellulose. In addition, the mechanism of liver toxicity of trimethylamine oxide (TOAM) involved in bacterial metabolism is still unclear.^[10]^

So far, there have been limited cohort-based studies investigating the causal relationship between GM and HCC. The timing of the occurrence of GM imbalance and HCC has not been definitively established in certain cross-sectional studies, thus hindering the determination of a causal relationship between them. Limited research has been conducted on the impact of diverse GM on liver cell impairment via distinct metabolic pathways. Although a causal relationship between GM and HCC has been reported in several studies,^[11]^ the underlying mechanisms of metabolic and immune abnormalities resulting from an imbalance in GM on HCC have not been extensively investigated.

Therefore, to address the above scientific challenges, this study conducted a Mendelian randomization (MR) association analysis of the gut microbiome and liver cancer occurrence, and tested metabolic products as potential mediator factors, aiming to explain the possible mechanism of HCC occurrence. The study collected the genome-wide association studies (GWAS) data from 3 Cohort studies (211 GM taxa, 1091 serum metabolites and 309 metabolite ratios and a HCC populations) and introduced MR analysis which similar to the randomized controlled trials design to interpret the causal relationship between HCC and related GM as well as the Intermediate factor (metabolites level or ratio). In this study, MR methodology was used to evaluate the causal effects of genetically proxied GM elements of interest on outcome-associated single nucleotide polymorphisms (SNPs) as instrumental variables (IVs) and related pathways to explain the mechanism.

## 2. Methods

### 2.1. Study design

**Figure 1.**
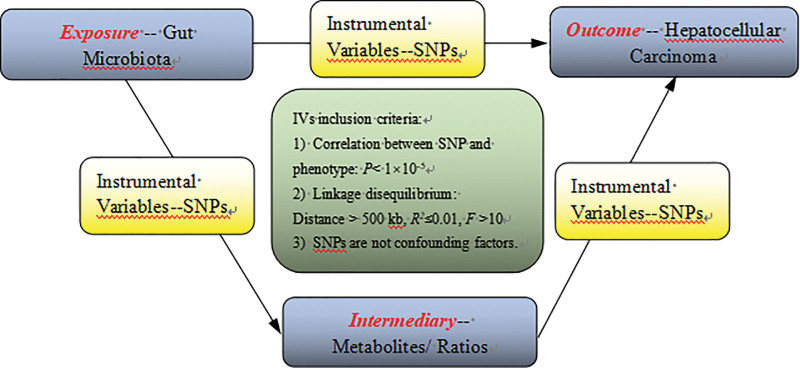
Schematic of the MR analysis. Significant IVs were selected to acssess the correlation between GM, metabolites and HCC. The 3 basic assumptions of MR analysis were illustrated in the acyclic graph. GM = gut microbiota, HCC = hepatocellular carcinoma, IVs = instrumental variables, MR = Mendelian randomization.

In the current study, we comprehensively evaluated the relationship between GM, metabolites and HCC from 3 populations one by one rigorously based on the MR design. A scientific MR study must include the testing of the following 3 hypotheses: genetic IVs are strongly associated with the exposure variables; genetic IVs should be irrelevant to the outcome variables and independent of any known or unknown confounding factors; and the effect of IVs on the results is mediated only by the exposure. Briefly, a causal analysis strategy was employed to select genetically significant SNPs for GM/metabolites and HCC. In order to avoid the sample overlap, genetic information of exposure intermediate factor and outcome were selected from independent GWAS datasets in this study. In this study, metabolites/ratios were screened as mediators, both as causative factors significantly associated with HCC and as outcome factors associated significantly with possible GM exposed microbiota. A schematic of this study is shown in Figure 1.

### 2.2. Ethics statement

The complete dataset utilized in this research is publicly accessible on an open website. The GWAS summary statistics have already been made available to the public through publication. Each institutional review board’s ethics committee granted written informed consent from all participants involved in separate cohort studies. No additional ethical approval or explicit consent was deemed necessary for this particular study.

### 2.3. GWAS data for GM

Based on the research conducted by Kurilshikov et al,^[12]^ submitted to the MiBioGen consortium (https://mibiogen.gcc.rug.nl/), we obtained 16S rRNA gene sequencing profiles and genotyping data from a total of 18,340 samples in order to investigate the relationship between genetic variation and GM. All participants included in the MiBioGen consortium were recruited from 25 cohorts across 11 countries. Through our GWAS study, we successfully identified a total of 122,110 variant sites within 211 taxa spanning from genus to the phylum level by analyzing GM taxa variation across diverse populations. From this extensive GWAS analysis, we extracted IVs representing GM taxa at 5 different taxonomic levels (Phylum, Class, Order, Family and Genus).

### 2.4. GWAS data for human serum metabolites/radio (potential intermediate compounds)

A genome-wide association aggregate dataset of 1091 human serum metabolites and 309 metabolite ratios involved in this study was obtained by Chen et al.^[13]^ These data are publicly available from the GWAS server (http://ftp.ebi.ac.uk/pub/databases/gwas/summary_statistics/GCST90199001-GCST90200000). The service platform has gathered comprehensive data on human serum metabolomics. The GWAS analysis encompassed a cohort of 8299 individuals from the Canadian Longitudinal Study on Aging. Through this analysis, we identified associations between 248 genetic loci and 690 metabolite levels, as well as 69 genetic loci and 143 metabolite ratios. By incorporating information on metabolite genes and the gene expression, we successfully identified 94 effector genes responsible for regulating the levels of 109 specific metabolites and 48 metabolite ratios. Furthermore, there are still approximately 241 unknown or partially characterized metabolites whose chemical properties remain to be fully determined.

### 2.5. GWAS data for HCC

The GWAS data of HCC among East Asia population were obtained from the data of the integrative epidemiology unit open GWAS project (https://gwas.mrcieu.ac.uk/). The GWAS ID of this dataset was bbj-a-158. In this GWAS Meta-analysis, the summary data included 1866 HCC cases and 195,745 control cases, yielding a total of 8885,115 SNPs. We extracted SNPs by analyzing visual component framework files shared by integrative epidemiology unit platform.

### 2.6. Selection of IVs

In this MR analysis, we employed 3 fundamental assumptions for the selection of IVs. Firstly, we established a genome-wide significance threshold of *P* <1 × 10^−5^ to identify highly associated SNPs for each metabolite. Secondly, we utilized an R software-based clumping procedure to detect independent variants by considering a linkage-disequilibrium avoidance criterion of *R^2^* <0.001 within a 500-kilobase (kb) distance. Thirdly, in order to quantitatively assess the strength of correlation between the selected SNPs and the exposure, we calculated both the phenotypic variation explained and *F*-statistic values. For subsequent operations, it is generally recommend considering a threshold of *F*-statistic >10.^[14]^

### 2.7. MR analysis

In this analysis, the prioritized evaluation approach for exploring the causal association between GM/metabolites and HCC was a standard method called inverse variance weighting (IVW). The IVW method is considered to be the most efficient MR method for estimating the causal effect of metabolite accurately when all IVs meet the 3 major hypotheses. However, inaccurate results may occur if some IVs do not adhere to these hypotheses. Hence, following sensitivity analyses were performed: *Q* tests were performed using the MR–Egger methods to detect heterogeneity between each instrument variable and the possibility of violating the assumption^[15]^; the MR–Egger intercept was used to estimate the horizontal pleiotropy, ensuring that the genetic variation was independently related to the exposure and outcome^[16]^; alternative methods, such as incorporating weighted median and MR-Egger techniques, were employed to improve the dependability and consistency of hypothesis testing; and The individual SNP analysis and leave-one-out test were used to compute the likelihood of relevance observed by individual SNP. To address potential confounding, after screening out qualified IVs (mutated gene loci), we had compared them with the human reference genome and eliminate those that have been confirmed to be significantly associated with risk factors (alcohol consumption, alcoholism and viral hepatitis) of the outcome variable.

### 2.8. Metabolic pathway analysis

Metabolome enrichment pathways associated with HCC were estimated using Web-based metconflict 5.0. (https://www.Metaboanalyst.ca/).^[17]^ The analysis modules for pathway and enrichment were utilized to identify potential clusters of metabolites or super-pathways that could be linked to metabolic processes and their potential association with HCC. The small molecule pathway database (SMPDB) and the Kyoto Encyclopedia of Genes and Genomes database were consulted as references. A significant level of 0.05 was considered for the enrichment pathway. This study identified several potential metabolic pathways associated with liver cancer through pathway enrichment analysis of influential metabolites. However, it should be noted that these findings only suggest a possible association between the pathways and liver cancer development, and the evidence for a causal relationship remains limited. Further experimental studies and cohort observations are required to validate these preliminary results.

### 2.9. Intermediate effect analysis

The MR method preserves the advantages of utilizing genetic instruments in establishing causal relationships, by mitigating bias caused by confounding factors and enabling estimation of distinct effects necessary for analysis of intermediate effect. The total effect (Beta_total_) is the effective value of GM on HCC; Beta_1_ is the effect value of GM on metabolites; Beta_2_ is the effect value of metabolites on HCC. Thus, the intermediate effect (Beta_12_) = Beta_1_ × Beta_2._ In addition, the direct effect (Beta_dir_) = Beta_total−_Beta_12_.^[18]^

### 2.10. Statistical analysis

The MR analyses were conducted using the “TwoSampleMR” package of R (version 4.3.1). Statistical significance was defined as *P* <.05, and the magnitude and direction of metabolic impact were estimated using odds ratios with corresponding 95% confidence intervals (95% CI). The sankey plot were drawn using Chiplot (https://www.chiplot.online/).

## 3. Results

### 3.1. Influence of GM on HCC

**Figure 2.**
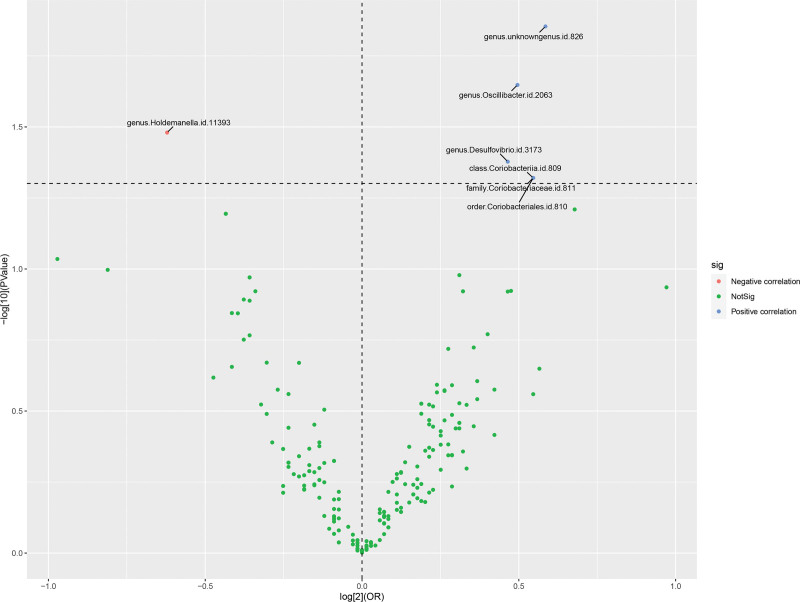
Volcano plot of correlations related to the influence of GM on HCC. This plot includes both ORs in-log 2 scale and *P*-values in -log 10 estimated by the inverse variance weighting method for HCC. GM = gut microbiota, HCC = hepatocellular carcinoma, OR = odds ratios.

Given a genome-wide significance threshold of *P* <1 × 10^−5^ for selecting highly associated SNPs among 211 GM, the filtered IVs comprised a total of 1839 SNPs, with a median count of 9 SNPs. The *F*-statistic values all exceeded 10, indicating the absence of significant instrumental bias. The primary analytical methodology employed in all MR analyses was IVW, with no observed heterogeneity and absence of weak IVs.^[19]^ The HCC dataset in this East Asian population was notably influenced by *Coriobacteriia* class, *Coriobacteriales* order, *Coriobacteriaceae* family, and 4 specific genera (*P* <.05 for IVW). Among them, *Holdemanella* genus was the protective factor (OR = 0.65, 95% CI: 0.43–0.97) and the other gut flora were the risk factors (Table 1 and Fig. 2).

**Table 1:** The 3 MR model estimates of the causal relationships between GM and HCC and tests for heterogeneity and horizontalpleiotropy.

GM	SNP (N)	Method	OR (95% CI)	*P*	Heterogeneity	*P*	Pleiotropy	*P*
					*Q* value		Intercept	
genus.unknowngenus.id.826	8	IVW	1.5 (1.09–2.08)	.0140	5.9221	.5489	−0.0087	.8583
		MR–Egger	1.66 (0.56–4.95)	.3974	5.8874	.4359	–	–
		WM	1.42 (0.91–2.23)	.1241	–	–	–	–
genus.*Oscillibacter*.id.2063	9	IVW	1.41 (1.05–1.9)	.0225	3.1146	.926962	0.0249	.7030
		MR–Egger	1.06 (0.25–4.48)	.9378	2.9568	.8890	–	–
		WM	1.32 (0.89–1.95)	.1672	–	–	–	–
genus.*Holdemanella*.id.11393	6	IVW	0.65 (0.43–0.97)	.0331	5.9432	.311789	−0.0196	.9389
		MR–Egger	0.79 (0.01–110.46)	.9314	5.9333	.2042	–	–
		WM	0.66 (0.4–1.09)	.1063	–	–	–	–
genus.*Desulfovibrio*.id.3173	9	IVW	1.38 (1.01–1.89)	.0419	7.2633	.508511	0.0387	.4514
		MR–Egger	0.92 (0.32–2.64)	.8756	6.6273	.4687	–	–
		WM	1.23 (0.81–1.87)	.3337	–	–	–	–
order.*Coriobacteriales*.id.810	15	IVW	1.46 (1–2.12)	.0478	16.7950	.267268	0.0078	.8820
		MR–Egger	1.29 (0.27–6.29)	.7537	16.7654	.2103	–	–
		WM	1.45 (0.87–2.4)	.1520	–	–	–	–
family.*Coriobacteriaceae*.id.811	15	IVW	1.46 (1–2.12)	.0478	16.7950	.267268	0.0078	.8820
		MR–Egger	1.29 (0.27–6.29)	.7537	16.7654	.2103	–	–
		WM	1.45 (0.87–2.41)	.1561	–	–	–	–
class.*Coriobacteriia*.id.809	15	IVW	1.46 (1–2.12)	.0478	16.7950	.267268	0.0078	.8820
		MR–Egger	1.29 (0.27–6.29)	.7537	16.7654	.2103	–	–
		WM	1.45 (0.88–2.37)	.1413	–	–	–	–

CI = confidence interval, IVW = inverse variance weighting, OR = odds ratio, SNP = single nucleotide polymorphism, WM **=** weighted median.

### 3.2. Influence of metabolites on HCC

**Figure 3.**
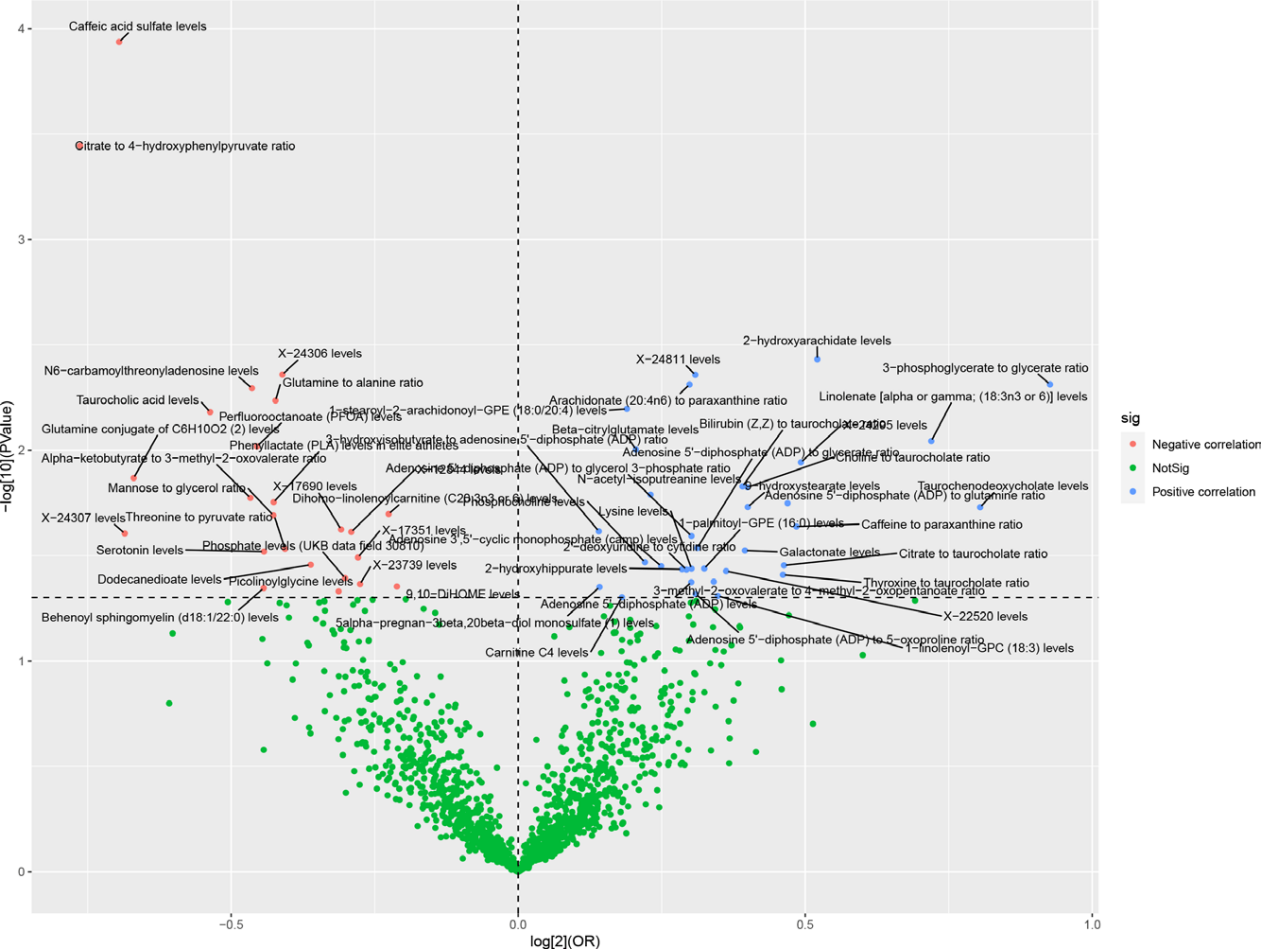
Volcano plot of correlations related to the influence of metabolite levels/ratios on HCC. This plot includes both ORs in-log 2 scale and *P*-values in -log 10 estimated by the inverse variance weighting method for HCC. HCC = hepatocellular carcinoma, OR = odds ratios.

Considering a genome-wide significance threshold of *P* <1 × 10^−5^ to select highly associated SNPs among 211 GM, the filtered IVs consisted of a total of 18,240 SNPs, with a median count of 12 SNPs. The *F*-statistic values consistently exceeded 10, indicating the absence of significant instrumental bias.The HCC dataset in East Asian population exhibited significant 49 influential metabolites (*P* <.05 for IVW), in which 31 were positively associated with HCC and 18 were negatively associated with HCC. Caffeic acid sulfate levels (*P* = .0001) was the most significant factor, followed by Citrate to 4-hydroxyphenylpyruvate ratio (*P* = .0004) and 2-hydroxyarachidate levels (*P *= .0037). Among these factors identified through Table 2 and Figure 3 analysis results.

**Table 2:** The 3 MR model estimates of the causal relationships between Metabolites and HCC and tests for heterogeneity and horizontalpleiotropy.

Metabolite	SNP (N)	Method	OR (95% CI)	*P*	Heterogeneity	*P*	Pleiotropy	*P*
					*Q* value		Intercept	
Caffeic acid sulfate levels	10	IVW	0.62 (0.48–0.79)	.0001	8.2805	.5061	0.0365	.5470
		MR–Egger	0.46 (0.18–1.18)	.1451	7.8850	.4448	–	–
		WM	0.58 (0.41–0.83)	.0024	–	–	–	–
Citrate to 4-hydroxyphenylpyruvate ratio	6	IVW	0.59 (0.44–0.79)	.0004	1.2394	.9410	−0.0328	.5721
		MR–Egger	0.79 (0.3–2.1)	.6603	0.8618	.9300	–	–
		WM	0.61 (0.43–0.88)	.0079	–	–	–	–
2-hydroxyarachidate levels	12	IVW	1.44 (1.12–1.83)	.0037	6.5732	.8325	−0.0193	.6597
		MR–Egger	1.68 (0.82–3.42)	.1865	6.3673	.7835	–	–
		WM	1.51 (1.08–2.11)	.0153	–	–	–	–
3-phosphoglycerate to glycerate ratio	7	IVW	1.9 (1.22–2.97)	.0049	0.7080	.9943	−0.0317	.7392
		MR–Egger	2.74 (0.34–22.05)	.3870	0.5841	.9887	–	–
		WM	1.85 (1.03–3.32)	.0385	–	–	–	–
Arachidonate (20:4n6) to paraxanthine ratio	7	IVW	1.23 (1.06–1.42)	.0049	6.4292	0.3769	−0.0007	0.9814
		MR–Egger	1.23 (0.95–1.6)	.1742	6.4284	.2667	–	–
		WM	1.24 (1.03–1.5)	.0223	–	–	–	–
N6-carbamoylthreonyladenosine levels	17	IVW	0.73 (0.58–0.91)	.0051	15.8507	.4634	−0.0613	.1627
		MR–Egger	1.37 (0.57–3.29)	.4942	13.6947	.5488	–	–
		WM	0.83 (0.6–1.15)	.2592	–	–	–	–
Glutamine to alanine ratio	19	IVW	0.75 (0.61–0.92)	.0058	14.9866	.6629	0.0439	.3263
		MR–Egger	0.49 (0.21–1.14)	.1149	13.9651	.6696		
		WM	0.69 (0.51–0.94)	.0192	–	–	–	–
1-stearoyl-2-arachidonoyl-GPE (18:0/20:4) levels	22	IVW	1.14 (1.04–1.25)	.0064	20.6449	.4808	−0.0105	.5342
		MR–Egger	1.2 (1.00–1.43)	.0618	20.2399	.4430	–	–
		WM	1.2 (1.06–1.36)	.0044	–	–	–	–
Taurocholic acid levels	11	IVW	0.69 (0.53–0.9)	.0066	6.7469	.7491	−0.0474	.7355
		MR–Egger	1.18 (0.06–24.23)	.9180	6.6255	.6760	–	–
		WM	0.68 (0.46–0.99)	.0465	–	–	–	–
Linolenate [alpha or gamma; (18:3n3 or 6)] levels	8	IVW	1.65 (1.13–2.4)	.0091	6.3751	.4967	−0.0538	.4736
		MR–Egger	3.06 (0.6–15.64)	.2278	5.7908	.4470	–	–
		WM	1.53 (0.92–2.54)	.1008	–	–	–	–
PFOA levels	11	IVW	0.73 (0.57–0.93)	.0096	3.5766	.9644	−0.0279	.5339
		MR–Egger	0.97 (0.39–2.39)	.9515	3.1583	.9577	–	–
		WM	0.68 (0.49–0.93)	.0171	–	–	–	–
Beta-citrylglutamate levels	17	IVW	1.15 (1.03–1.28)	.0099	13.9658	0.6013	0.0065	0.7227
		MR–Egger	1.12 (0.93–1.35)	.2422	13.8351	.5381	–	–
		WM	1.11 (0.96–1.27)	.1484	–	–	–	–
Glutamine conjugate of C6H10O2 (2) levels	7	IVW	0.63 (0.43–0.91)	.0136	6.0162	.4214	−0.0953	.5040
		MR–Egger	2.1 (0.08–57.08)	.6788	5.4516	.3633	–	–
		WM	0.61 (0.38–0.99)	.0434	–	–	–	–
Bilirubin (Z, Z) to taurocholate ratio	9	IVW	1.31 (1.05–1.63)	.0149	3.8622	.8693	0.0151	.7240
		MR–Egger	1.18 (0.65–2.16)	.6070	3.7270	.8106	–	–
		WM	1.22 (0.91–1.64)	.1755	–	–	–	–
Choline to taurocholate ratio	11	IVW	1.32 (1.06–1.65)	.0150	7.8509	.6434	0.0136	.7036
		MR–Egger	1.17 (0.61–2.23)	.6490	7.6966	.5650	–	–
		WM	1.31 (0.94–1.83)	.1066	–	–	–	–
N-acetyl-isoputreanine levels	21	IVW	1.17 (1.03–1.34)	.0162	15.2368	.7627	−0.0220	.2567
		MR–Egger	1.34 (1.03–1.74)	.0394	13.8695	.7912	–	–
		WM	1.24 (1.04–1.48)	.0168	–	–	–	–
Alpha-ketobutyrate to 3-methyl-2-oxovalerate ratio	12	IVW	0.72 (0.56–0.94)	.0168	11.9640	.3664	−0.0523	.2747
		MR–Egger	1.24 (0.48–3.18)	.6694	10.5542	.3933	–	–
		WM	0.86 (0.59–1.25)	.4385	–	–	–	–
PLA levels in elite athletes	15	IVW	0.74 (0.58–0.95)	.0176	14.4782	.4147	0.0358	.2753
		MR–Egger	0.53 (0.28–0.99)	.0698	13.1645	.4352	–	–
		WM	0.74 (0.53–1.01)	.0613	–	–	–	–
ADP to glutamine ratio	12	IVW	1.38 (1.06–1.81)	.0179	14.4826	0.2074	−0.2493	0.0162
		MR–Egger	13.35 (2.81–63.33)	.0085	6.1516	.8024	–	–
		WM	1.4 (1–1.96)	.0512	–	–	–	–
9-hydroxystearate levels	12	IVW	1.32 (1.05–1.66)	.0186	8.4665	.6710	0.0614	.1266
		MR–Egger	0.83 (0.46–1.5)	.5522	5.6895	.8406	–	–
		WM	1.28 (0.91–1.8)	.1520	–	–	–	–
Taurochenodeoxycholate levels	6	IVW	1.75 (1.1–2.78)	.0187	1.0968	.9544	−0.0180	.8081
		MR–Egger	2.1 (0.49–8.97)	.3747	1.0295	.9053	–	–
		WM	1.71 (0.96–3.04)	.0680	–	–	–	–
Dihomo-linolenoylcarnitine (C20:3n3 or 6) levels	23	IVW	0.86 (0.75–0.98)	.0201	18.9455	.6487	0.0060	.7387
		MR–Egger	0.82 (0.63–1.07)	.1600	18.8313	.5960	–	–
		WM	0.87 (0.72–1.05)	.1418	–	–	–	–
Mannose to glycerol ratio	11	IVW	0.74 (0.58–0.96)	.0204	10.4692	.4003	0.0347	.4354
		MR–Egger	0.54 (0.24–1.21)	.1678	9.7476	.3713	–	–
		WM	0.72 (0.51–1.01)	.0584	–	–	–	–
Caffeine to paraxanthine ratio	11	IVW	1.4 (1.05–1.87)	.0230	11.9462	.2887	0.0804	.2036
		MR–Egger	0.64 (0.2–2.03)	.4648	9.8823	.3601	–	–
		WM	1.08 (0.74–1.57)	.6983	–	–	–	–
3-hydroxyisobutyrate to ADP ratio	15	IVW	1.1 (1.01–1.2)	.0242	12.9066	.5339	−0.0175	.3754
		MR–Egger	1.15 (1.02–1.3)	.0463	12.0643	.5224	–	–
		WM	1.12 (1.01–1.25)	.0308	–	–	–	–
ADP to glycerol 3-phosphate ratio	14	IVW	1.23 (1.03–1.48)	.0255	19.3863	0.1116	0.0133	0.6740
		MR–Egger	1.14 (0.76–1.7)	.5327	19.0906	.0864	–	–
		WM	1.24 (0.99–1.54)	.0574	–	–	–	–
ADP to glycerate ratio	12	IVW	1.24 (1.02–1.51)	.0291	5.2563	.9181	0.0374	.3223
		MR–Egger	0.94 (0.53–1.65)	.8263	4.1721	.9393	–	–
		WM	1.13 (0.88–1.45)	.3376	–	–	–	–
Threonine to pyruvate ratio	15	IVW	0.75 (0.59–0.97)	.0294	19.9332	.1323	0.1185	.0539
		MR–Egger	0.24 (0.08–0.71)	.0231	14.8169	.3189	–	–
		WM	0.8 (0.57–1.11)	.1788	–	–	–	–
Galactonate levels	6	IVW	1.31 (1.03–1.68)	.0299	1.6316	.8974	0.0186	.7330
		MR–Egger	1.17 (0.6–2.28)	.6642	1.4977	.8271	–	–
		WM	1.27 (0.92–1.76)	.1451	–	–	–	–
Serotonin levels	13	IVW	0.74 (0.56–0.97)	.0303	7.1073	.8504	0.0415	.6130
		MR–Egger	0.47 (0.09–2.56)	.4030	6.8364	.8122	–	–
		WM	0.72 (0.5–1.02)	.0676	–	–	–	–
Phosphocholine levels	20	IVW	1.17 (1.01–1.34)	.0340	15.6028	.6836	−0.0118	.6373
		MR–Egger	1.27 (0.88–1.83)	.2235	15.3728	.6362	–	–
		WM	1.1 (0.91–1.32)	.3323	–	–	–	–
Dodecanedioate levels	16	IVW	0.78 (0.62–0.98)	.0350	18.4612	.2392	0.0183	.6551
		MR–Egger	0.65 (0.29–1.45)	.3113	18.1906	.1982	–	–
		WM	0.8 (0.57–1.14)	.2127	–	–	–	–
Citrate to taurocholate ratio	5	IVW	1.38 (1.02–1.86)	.0352	3.9055	0.4189	0.0198	0.7109
		MR–Egger	1.17 (0.51–2.72)	.7326	3.7005	.2957	–	–
		WM	1.36 (0.92–2.01)	.1260	–	–	–	–
Adenosine 3’,5’-cyclic monophosphate (camp) levels	18	IVW	1.19 (1.01–1.4)	.0355	22.4565	.1678	0.0028	.9223
		MR–Egger	1.17 (0.78–1.75)	.4703	22.4428	.1295	–	–
		WM	1.16 (0.93–1.45)	.1884	–	–	–	–
1-palmitoyl-GPE (16:0) levels	11	IVW	1.25 (1.01–1.55)	.0364	13.0300	.2220	−0.0094	.8436
		MR–Egger	1.34 (0.67–2.67)	.4272	12.9706	.1639	–	–
		WM	1.34 (1.06–1.7)	.0148	–	–	–	–
Lysine levels	18	IVW	1.23 (1.01–1.5)	.0364	12.2350	.7857	0.0224	.4428
		MR–Egger	1.04 (0.65–1.66)	.8735	11.6157	.7700	–	–
		WM	1.22 (0.93–1.62)	.1564	–	–	–	–
2-hydroxyhippurate levels	14	IVW	1.22 (1.01–1.47)	.0367	7.1873	.8922	0.0225	.4222
		MR–Egger	1.04 (0.68–1.58)	.8603	6.4967	.8890	–	–
		WM	1.2 (0.93–1.56)	.1671	–	–	–	–
2’-deoxyuridine to cytidine ratio	12	IVW	1.23 (1.01–1.48)	.0368	4.9826	.9320	−0.0191	.5112
		MR–Egger	1.39 (0.92–2.12)	.1501	4.5184	.9209	–	–
		WM	1.12 (0.85–1.46)	.4303	–	–	–	–
Thyroxine to taurocholate ratio	8	IVW	1.38 (1.02–1.86)	.0390	10.5900	.1575	−0.0116	.8349
		MR–Egger	1.52 (0.58–3.96)	.4230	10.5070	.1049	–	–
		WM	1.56 (1.08–2.25)	.0173	–	–	–	–
Phosphate levels (UKB data field 30,810)	15	IVW	0.81 (0.66–0.99)	.0404	15.6559	0.3348	0.0035	0.8873
		MR–Egger	0.8 (0.57–1.12)	.2077	15.6307	.2696	–	–
		WM	0.86 (0.65–1.13)	.2657	–	–	–	–
3-methyl-2-oxovalerate to 4-methyl-2-oxopentanoate ratio	14	IVW	1.27 (1.01–1.59)	.0420	15.0876	.1785	−0.0321	.3196
		MR–Egger	1.61 (0.97–2.65)	.0925	13.5965	.1922	–	–
		WM	1.28 (0.97–1.71)	.0857	–	–	–	–
ADP levels	12	IVW	1.23 (1.01–1.51)	.0423	18.6814	.1333	0.0146	.7695
		MR–Egger	1.12 (0.57–2.20)	.7560	18.5427	.1002	–	–
		WM	1.17 (0.91–1.50)	.2306	–	–	–	–
9,10-DiHOME levels	14	IVW	0.86 (0.75–1.00)	.0443	11.5103	.4016	0.0009	.9668
		MR–Egger	0.86 (0.68–1.09)	.2372	11.5082	.3193	–	–
		WM	0.86 (0.70–1.06)	.1484	–	–	–	–
5alpha-pregnan-3beta,20beta-diol monosulfate (1) levels	20	IVW	1.10 (1.00–1.21)	.0446	17.8396	.5332	−0.0054	.7272
		MR–Egger	1.13 (0.97–1.3)	.1312	17.7141	.4746	–	–
		WM	1.12 (0.99–1.26)	.0761	–	–	–	–
Behenoyl sphingomyelin (d18:1/22:0) levels	9	IVW	0.74 (0.54–0.99)	.0451	7.1764	.5177	−0.0168	.7554
		MR–Egger	0.87 (0.3–2.53)	.8072	7.0704	.4216	–	–
		WM	0.91 (0.59–1.38)	.6444	–	–	–	–
Picolinoylglycine levels	13	IVW	0.81 (0.65–1)	.0467	11.9798	.4473	−0.0100	.6851
		MR–Egger	0.86 (0.58–1.28)	.4842	11.7939	.3793	–	–
		WM	0.81 (0.6–1.08)	.1464	–	–	–	–
ADP to 5-oxoproline ratio	9	IVW	1.24 (1–1.53)	.0481	9.1024	0.3337	0.0839	0.0778
		MR–Egger	0.76 (0.46–1.26)	.3210	4.8402	.6795	–	–
		WM	1.18 (0.88–1.6)	.2708	–	–	–	–
1-linolenoyl-GPC (18:3) levels	13	IVW	1.27 (1–1.62)	.0491	13.2316	.3524	−0.0196	.5981
		MR–Egger	1.54 (0.74–3.18)	.2702	12.8865	.3008	–	–
		WM	1.37 (0.98–1.91)	.0631	–	–	–	–
Carnitine C4 levels	24	IVW	1.13 (1–1.28)	.0499	25.7675	.3120	0.0277	.1046
		MR–Egger	0.97 (0.79–1.21)	.8042	22.7982	.4132	–	–
		WM	1.04 (0.87–1.24)	.6933	–	–	–	–

ADP = adenosine 5’-diphosphate, CI = confidence interval, IVW **=** inverse variance weighting, MR = Mendelian randomization, OR = odds ratio, PFOA = perfluorooctanoate, PLA = phenyllactate, SNP = single nucleotide polymorphism, WM **=** weighted median.

**Figure 4.**
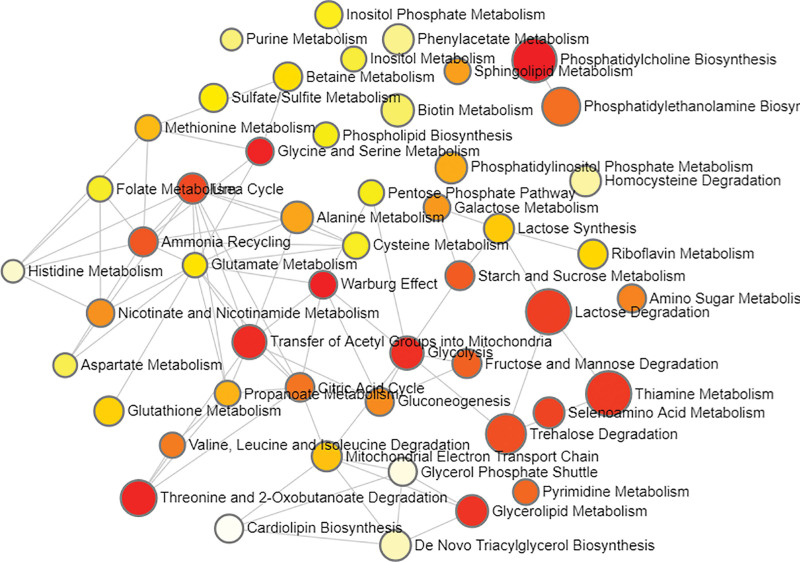
Network of enrichment pathways of the metabolites selected by MR of meatabolite levels/ratios on HCC. The color ranges from light yellow to dark red, indicating the level of enrichment significance, and the size of the circle reflects the level of the enrichment ratio. HCC = hepatocellular carcinoma.

The metabolites significantly associated with HCC were analyzed using the Metabolic Analyzer 5.0 platform to elucidate the underlying metabolic pathways implicated in HCC pathogenesis. The metabolites associated with HCC were significantly enriched in 30 metabolic pathways (*P* <.05), as evidenced by a thorough comparison with the small molecule pathway database (SMPBD). Table 3 presents the top 10 most significant pathways, among which Phosphatidylcholine Biosynthesis involving Choline, Adenosine 5’-diphosphate (ADP), and Phosphorylcholine stands out as the most prominent enrichment pathway(*P* = .0019). ADP and Phosphate are important metabolites in almost all enrichment pathways. Figure 4 illustrates the relationship network of each metabolic pathway.

**Table 3:** The top 10 significant enrichment pathways of the metabolites selected by MR.

Pathway	Total metabolites	Metabolites involved in enrichment	*P*-value	FDR
Phosphatidylcholine biosynthesis	14	Choline, ADP, phosphorylcholine	.0019	0.103
Warburg effect	57	Citric acid, glutamine, 3-phosphoglyceric acid, ADP, phosphate	.0031	0.103
Glycine and serine metabolism	59	2-ketobutyric acid, L-threonine, 3-phosphoglyceric acid, ADP, phosphate	.0037	0.103
Threonine and 2-oxobutanoate degradation	20	2-ketobutyric acid, L-threonine, ADP	.0054	0.103
Transfer of acetyl groups into mitochondria	22	Citric acid, ADP, phosphate	.0071	0.103
Glycolysis	23	3-phosphoglyceric acid, ADP, phosphate	.0081	0.103
Glycerolipid metabolism	25	3-phosphoglyceric acid, ADP, phosphate	.0102	0.103
Thiamine metabolism	9	ADP, phosphate	.0113	0.103
Lactose degradation	9	ADP; phosphate	.0113	0.103
Selenoamino acid metabolism	27	2-ketobutyric acid, ADP, phosphate	.0127	0.103

ADP = adenosine 5’-diphosphate, FDR = false discovery rate, MR = Mendelian randomization.

### 3.3. Screening of intermediary factor affecting HCC by GM

**Figure 5.**
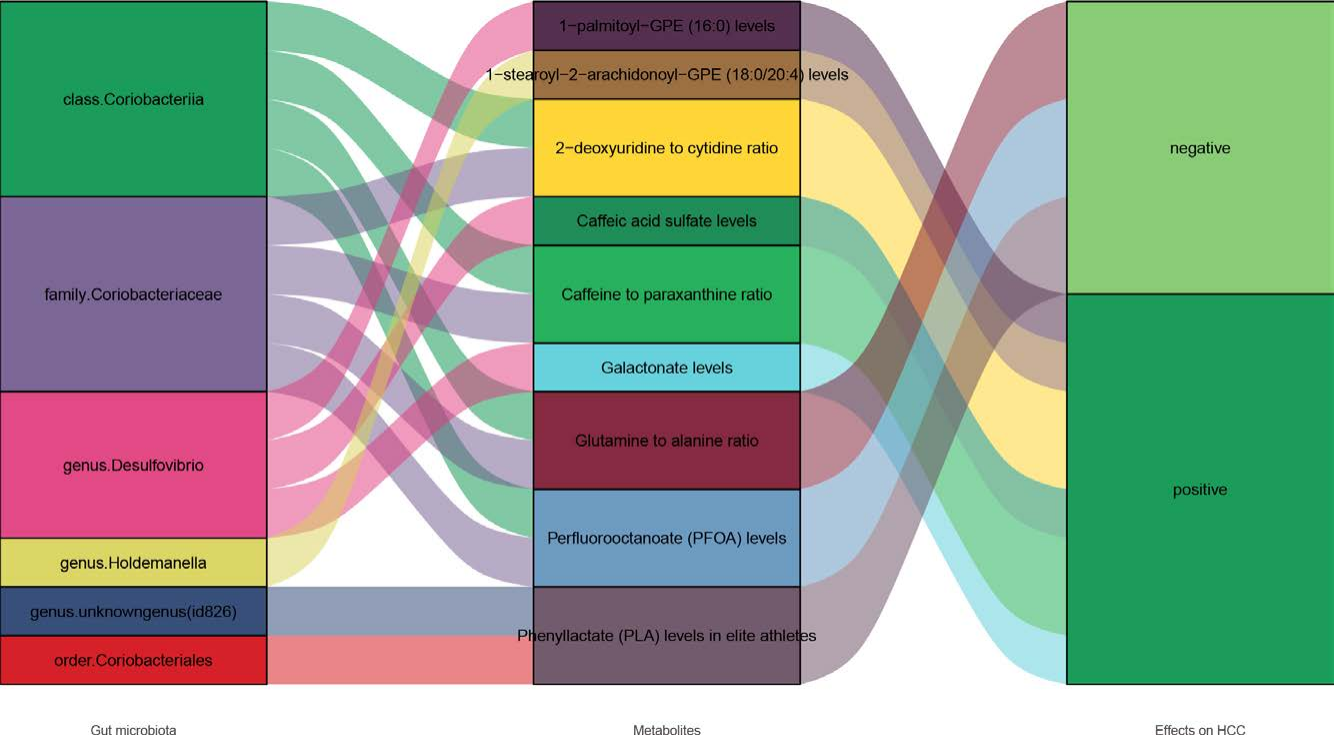
Sankey plot of GM, Metabolite levels/ratios and HCC. GM are the exposure factors, metabolite levels/ratios are the intermediate factors and HCC is the outcome factors. GM = gut microbiota, HCC = hepatocellular carcinoma.

Intermediary factor analysis was used to analyze the bridging effect of metabolites on GM and HCC. MR Analysis was employed for intermediary factors screening. The IVW causal association model was established for 7 GM that significantly influenced HCC development and 49 named metabolites associated with HCC. Finally, 10 intermediate metabolites levels or ratios were selected by IVW method, of which 5 were positively correlated and 5 were negatively correlated. Coriobacteriaceae family and class share 4 metabolites (2’-deoxyuridine to cytidine ratio, Perfluorooctanoate (PFOA) levels, Glutamine to alanine ratio, Caffeine to paraxanthine ratio). The Coriobacteriales order and Unknown genus (id: 826) shared Phenyllactate (PLA) levels in elite athletes. *Desulfovibrio genus* had 3 metabolites (1-palmitoyl-GPE (16:0) levels, Caffeic acid sulfate levels, Galactonate levels). The mediator of the Holdemanella genus was 1-stearoyl-2-arachidonoyl-GPE (18:0/20:4) levels. (Table 4 and Fig. 5.)

**Table 4:** Intermediary factor analysis between GM, metabolites and HCC using the IVW MR approach.

GM (exposure)	Beta_total_	Beta _1_	OR (95% CI)	Metabolites (intermediary factor)	Beta _2_	OR (95% CI)	Beta_12_	Beta_dir_
class.*Coriobacteriia* and family.*Coriobacteriaceae*	0.3767	−0.2007	0.82 (0.7–0.96)	2’-deoxyuridine to cytidine ratio	0.2032	1.23 (1.01–1.48)	−0.0408	0.4175
		0.2059	1.23 (1.04–1.45)	PFOA levels	−0.3154	0.73 (0.57–0.93)	−0.0649	0.4416
		−0.1608	0.85 (0.73–0.99)	Glutamine to alanine ratio	−0.2930	0.75 (0.61–0.92)	0.0471	0.3296
		0.1578	1.17 (1–1.37)	Caffeine to paraxanthine ratio	0.3360	1.4 (1.05–1.87)	0.0530	0.3237
genus.*Desulfovibrio*	0.3240	0.1877	1.21 (1.05–1.39)	1-palmitoyl-GPE (16:0) levels	0.2247	1.25 (1.01–1.55)	0.0422	0.2818
		−0.1697	0.84 (0.73–0.98)	Caffeic acid sulfate levels	−0.4818	0.62 (0.48–0.79)	0.0818	0.2422
		−0.1690	0.84 (0.73–0.98)	Galactonate levels	0.2738	1.31 (1.03–1.68)	−0.0463	0.3703
genus.*Holdemanella*	−0.4352	−0.13957762	0.87 (0.77–0.98)	1-stearoyl-2-arachidonoyl-GPE (18:0/20:4) levels	0.1313	1.14 (1.04–1.25)	−0.0183	−0.4169
order.*Coriobacteriales*	0.3767	0.12632496	1.13 (1–1.29)	PLA levels in elite athletes	−0.2952	0.74 (0.58–0.95)	−0.0373	0.4140
genus.unknowngenus (id826)	0.4082							0.4082

CI = confidence interval, GM = gut microbiota, GPE = glycerophosphoethanolamine, HCC = hepatocellular carcinoma, IVW = inverse variance weighting, MR = Mendelian randomization, OR = odds ratio, PFOA = perfluorooctanoate, PLA = phenyllactate.

* Beta_total_ is the effect value of GM on HCC; Beta_1_ is the effect value of GM on metabolites; Beta_2_ is the effect value of metabolites on HCC. Beta_12_ (Intermediate effect) = Beta_1_ × Beta_2_; Beta_dir_ (direct effect) = Beta_total_-Beta_12_.

**Figure 6.**
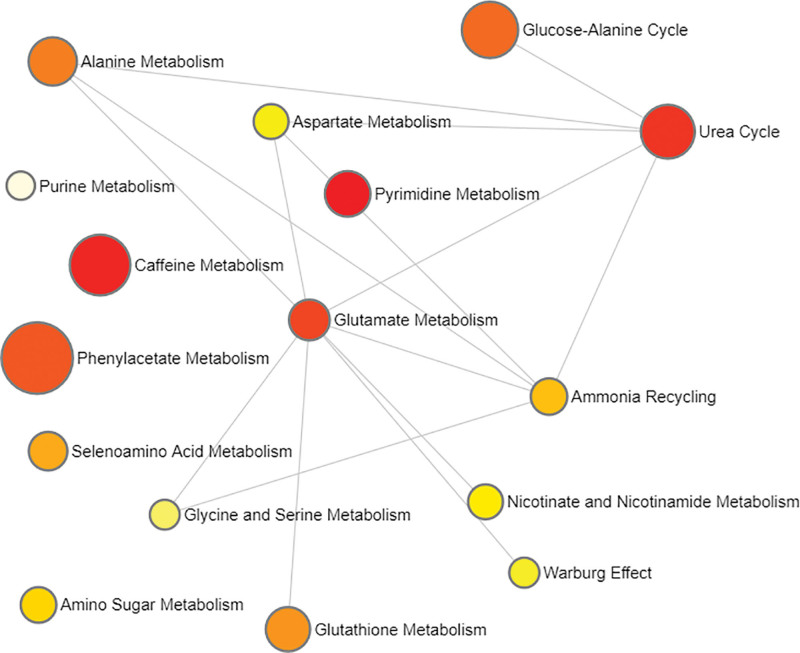
Network of enrichment pathways of the metabolites selected by intermediary factors screening. The color ranges from light yellow to dark red, indicating the level of enrichment significance, and the size of the circle reflects the level of the enrichment ratio.

The metabolic pathway enrichment of 10 metabolites levels and ratios found 4 significantly enriched metabolic pathways. The involvement of Deoxyuridine, Cytidine and Glutamine in Pyrimidine Metabolism was the most significant (*P *= .0031), followed by Caffeine Metabolism (*P *= .0072), in which Caffeine and Paraxanthine participate. The significance of Urea Cycle and Glutamate Metabolism co-participated by Glutamine and L-Alanine were 0.0105 and 0.0298, respectively. From the interaction network of enrichment pathways shown in Figure 6, it can be seen that Glutamate Metabolism is a key metabolic pathway node.

## 4. Discussion

Our study found *Coriobacteriia* class, *Coriobacteriales* order, *Coriobacteriaceae* family, and 4 specific genera were strongly related to HCC. Meanwhile, through 2 samples-MR, 49 metabolites levels/ratios were shown to be closely related to the development of HCC. In order to explore the metabolic mediators of GM’s influence on HCC, we used 7 GM taxa significantly associated with HCC as exposure, 49 metabolite levels/ratios as outcomes, and again performed 2 samples-MR. A total of 10 related metabolites were selected, and 4 metabolic pathways of Pyrimidine, Caffeine, Urea Cycle and Glutamate were significantly enriched. These results proved the mechanism of these 4 metabolic pathways in the process of liver damage caused by *Coriobacteriia* class and its lower taxa.

The *Coriobacteriia* class is the most significant group among the screened GMS that have been identified as being strongly associated with HCC. The order, family, and a similar unnamed genus under *Coriobacteriia*’s taxonomic rank are all significantly associated with HCC. The members of *Coriobacteriia* category comprise anaerobic organisms that thrive in diverse ecological environments and lack the capacity for spore production. They can be classified as either obligate or facultative based on their anaerobic requirements.^[20,21]^ Although studies on the correlation between Coriobacteriia and liver cancer are still limited, pathogens of this class are closely related to many human diseases. A recent study has reported a significant enrichment of *Coriobacteriaceae* and *Coriobacteriales* in the urethral secretions of patients diagnosed with chronic prostatitis.^[22]^ This particular type of urinary system infection was considered to be the main complication of cirrhosis,^[23]^ which undoubtedly speeds up the process from cirrhosis to HCC. Several studies have indicated that *Coriobacteriales* could potentially serve as a mediator in the process through which primary biliary cholangitis is triggered by smoking or exposure to environmental factors.^[24,25]^ In a Mendelian randomized study investigating the association between GM and fatty liver, Coriobacteriia emerged as a significant risk factor (*OR* = 1.22, 95% *CI* = 1.01–1.42),^[26]^ aligning with the directional hypothesis of our investigation. However, surprisingly, there was a decrease in the abundance of genera belonging to the *Coriobacteriia* class among familial hypercholesterolemia patients who had been on statin therapy for over 12 months compared to their healthy counterparts.^[27]^ Certainly, there have been reports indicating that the presence of class *Coriobacteriia* and its subsequent taxonomic levels, including order *Coriobacteriales* and family *Coriobacteriaceae*, exhibited a beneficial impact on allergic rhinitis.^[28]^

Earlier research has indicated that the prevalence of *Coriobacteriaceae* is notably elevated in mice suffering from nonalcoholic steatohepatitis, and this impact can be alleviated through the administration of ursodeoxycholic acid.^[29]^ It is obvious that ursodeoxycholic acid is likely to be one of the metabolites involved in the liver diseases caused by the microorganisms of *Coriobacteriaceae*. Our study provides a new way for gut microbes to cause liver cancer through metabolic pathways. In this study, 3 metabolites (Deoxyuridine, Cytidine and Glutamine) related to Coriobacteriia were significantly enriched in the pyrimidine metabolic pathway (*P* = .0031). The literature has also confirmed the influence of pyrimidine metabolism on HCC.^[30–32]^ uracil-cytidine kinase 2, a key regulator of pyrimidine metabolism, is elevated during the development of HCC and shows carcinogenic effects. The expression of uracil-cytidine kinase 2 was up regulated in part by TGF-βstimulation.^[33,34]^ However, how *Coriobacteriia* changes the intestinal environment and stimulates and regulates Cytidine remains to be further studied. Another significantly enriched pathway is caffeine metabolism (*P* = .0072). An observational study showed changes in GM following fat loss surgery, along with changes in caffeine metabolism.^[35]^ Numerous studies have shown that caffeine metabolism is closely related to liver disease.^[36–39]^ There were also evidences of enrichment the significance of Urea Cycle (*P* = .0105) and Glutamate Metabolism (*P* = .0298) co-participated by Glutamine and L-Alanine. Studies have shown that in HCC patients, the expression of genes in the urea cycle is continuously inhibited.^[40,41]^ From the interaction network of enrichment pathways, it can be seen that Glutamate Metabolism is a key metabolic pathway node. In a mouse xenograft models, oxoglutarate dehydrogenase-like (OGDHL) drove reduced carboxylation of glutamine-derived α-ketoglutaric (α-KG) acid in hepatocellular carcinoma cells by up-regulating the citric acid ratio of α-KG through retrograde tricarboxylic acid cycling.^[42]^ It indicated that the sensitivity of HCC cells to targeted therapy could be enhanced by reducing the metabolism of glutamine.

Another genus that affects HCC and has a mediating effect is *Desulfovibrio*. Research by Zhang et al showed an increased proportion of *Desulfovibrio genus* in mice with high cholesterol and fatty liver, which was corroborated in human hypercholesteremia patients.^[43]^ Another study based on a mouse model shows a positive correlation between *Desulfovibrio* levels and levels of bacterial lipopolysaccharides (LPS) derived from liver cells, suggesting the abnormal strain may be harmful to the liver.^[44]^ The aforementioned conclusions are in alignment with the findings of this study. 1-palmitoyl-glycerophosphoethanolamine (GPE) (16:0) levels, Caffeic acid sulfate levels and Galactonate levels were intermediate metabolite in this pathway. A transcriptomics and (13) C-isotope based metabolomics research showed that GPE level was significant with HCC (*P* = .015).^[45]^ Caffeic acid sulfate works by blocking the production of ROS (reactive oxygen species), inducing DNA oxidation in cancer cells, and reducing angiogenesis in tumor cells.^[46]^ Disturbances in galactose metabolism may result in the accumulation of this carbohydrate, potentially leading to damage in the brain and liver. And evidence from a study suggested that acute galactose administration could impair redox homeostasis in brain and liver of rats.^[47]^

In this study, the *Holdemanella* genus was regulated by 1-stearoyl-2- arachidonoyl-GPE (18:0/20:4) and showed a protective effect against HCC. Although the GM of patients with liver fibrosis is rich in immunogenic symbiotic bacteria, including *Holdemanella*,^[48]^ whether it has a positive or negative effect on liver disease progression remains to be explored. Interestingly, Phosphatidylethanolamine (PE) (18:0/20:4) was negatively associated with HCC and mediates its effect on decreasing HCC. PE, as the second most abundant glycerol phospholipid in eukaryotic cells, plays an indispensable role in various cellular processes.^[49]^

In addition to the GM, this study also analyzed 1400 metabolome responses to HCC. Phosphatidylcholine (PC) Biosynthesis involving Choline, Adenosine 5’-diphosphate (ADP), and PC stands out as the most prominent enrichment pathway(*P* = .0019). Lipidomics analysis revealed that the overexpression of SLC27A4 significantly enhanced the specific uptake of monounsaturated fatty acids (MUFAs), resulting in elevated levels of MUFA-containing phosphatidylcholine and phosphatidylethanolamine within HCC cells. Consequently, this led to increased resistance against lipid peroxidation and iron-induced apoptosis.^[50]^ However, another study has shown that PC was a protective factor in nonalcoholic steatohepatitis.^[51]^ Therefore, further research evidence regarding the impact of PC on hepatic function should be conducted based on clinical practice.

There are certain limitations in our study. Firstly, since the influence of GM on HCC is not limited to the regulation of metabolites, immune mechanisms may become confounding factors. Therefore, our next step will be to combine more omics information to conduct a comprehensive causal association and mediating effects study. Secondly, the GM lineage involved in this study needs to be further expanded to mine more clinically meaningful information. Thirdly, the development and progression of liver cancer may also be influenced by the intestinal microenvironment and metabolism, and the speed of its development varies. However, this study only obtained data on liver cancer diagnosis, making it difficult to conduct an analysis of the influencing factors of disease stage and treatment effect. In the future, cohort observations will be considered to be carried out in order to grasp the changing patterns and mechanisms of the microbiome and metabolome throughout the entire process of HCC development. Finally, there is a need for additional investigation into an unidentified Gunus (id 826), as it may hold significant medical implications.

## 5. Conclusion

This study found that 7 GM taxa were associated with HCC pathogenesis, and 6 of them affected HCC through metabolic intermediary pathways. Coriobacteriia class and its lower taxa were associated with the risk factors of developing HCC through the regulation of Pyrimidine, Caffeine, Urea Cycle and Glutamate metabolic pathways. These GM taxa and metabolitesmay serve as potential targets for the diagnosis of HCC.

## Acknowledgments

We are grateful to all the participants and investigators of the study, as well as to all investigators who contributed to the GWAS of modifiable risk factors.

## Author contributions

**Conceptualization:** Jingyi Dai, Yu Luo, Guiming Liu.

**Data curation:** Jingyi Dai, Qiujing Li, Jie Chen.

**Formal analysis:** Jingyi Dai, Qiujing Li, Jie Chen, Zhijian Dong, Zhongxu Ma.

**Funding acquisition:** Jingyi Dai, Yu Luo, Guiming Liu.

**Writing – original draft:** Jingyi Dai, Qiujing Li, Jie Chen, Zhijian Dong, Zhongxu Ma.

**Writing – review & editing:** Yu Luo, Guiming Liu.
